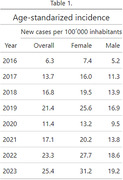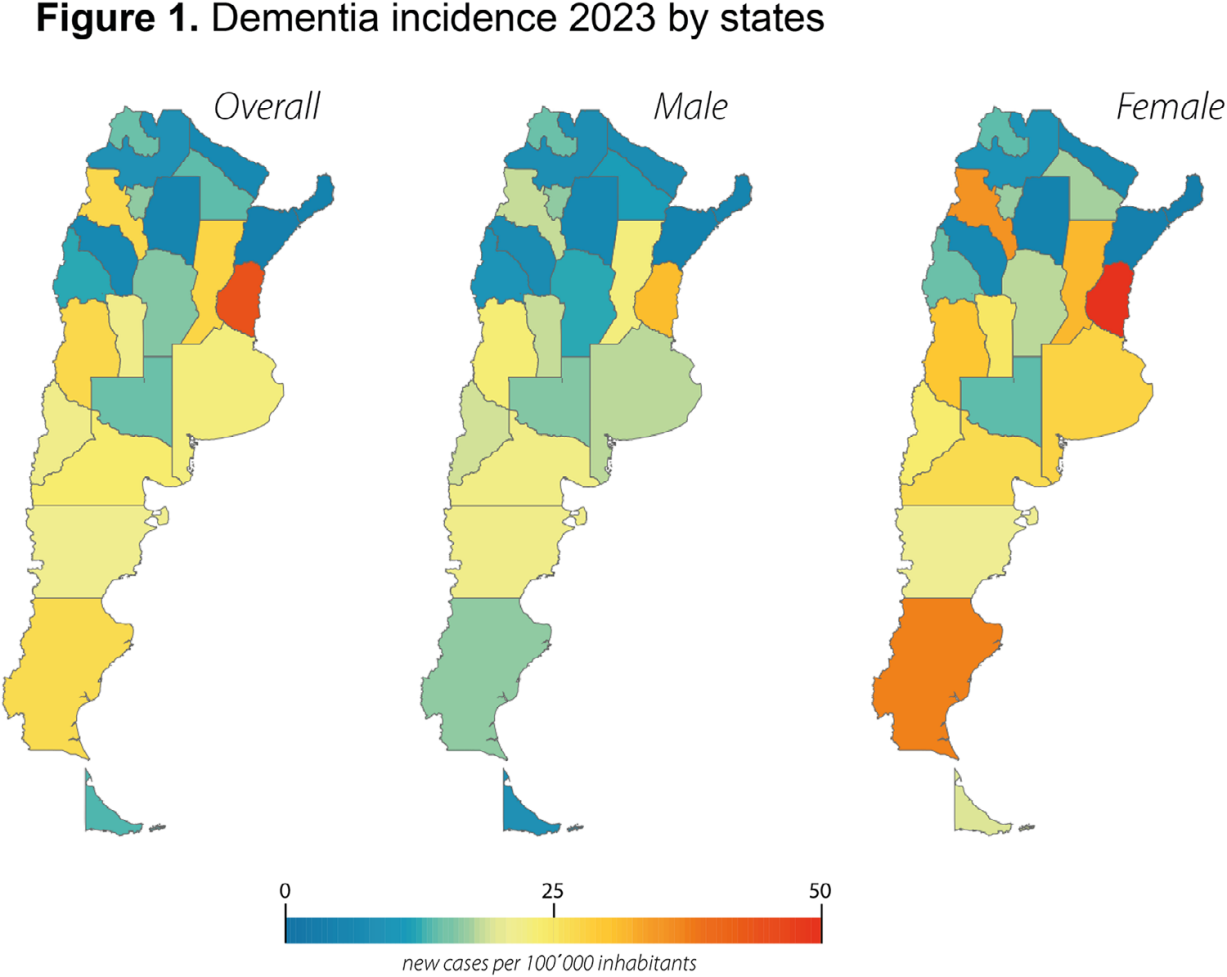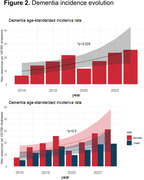# A Decade‐Long Analysis of Dementia Incidence in Argentina: Insights from a Public Registry Study

**DOI:** 10.1002/alz.090532

**Published:** 2025-01-09

**Authors:** Ismael Luis Calandri, Claudia Kimie Suemoto, Ricardo Allegri, Equipo de Estadística e Investigación social, Agencia Nacional de Discapacidad, Argentina

**Affiliations:** ^1^ Fleni, Buenos Aires, Buenos Aires Argentina; ^2^ Alzheimer Center Amsterdam, Amsterdam UMC, Amsterdam Netherlands; ^3^ University of São Paulo Medical School, São Paulo, São Paulo Brazil

## Abstract

**Background:**

Argentina is the second‐largest country in Latin America, home to 9% of the world’s Latino population. Like the rest of Latin America, it is grappling with the aging of its population and an increase in risk factors. Despite this, there are currently no studies on the incidence of dementia in the national territory. This study aims to present the current incidence rates and changes over the past decade for dementia in Argentina. Additionally, we explore sex‐specific and early‐onset dementia trends.

**Methods:**

Utilizing 10‐year data from the “National Disability Registry,” a comprehensive national database offering specialized health coverage, we identified cases certified by an interdisciplinary board. Dementia subjects were defined as those with mental disability onset post‐18 years, with etiological diagnosis as per the ICD‐10 code for dementia causes. We calculated age‐standardized incidence rates per 100,000 inhabitants using population projections based on the 2010 and 2022 national censuses. To explore incidence time trends, we fitted linear models to predict incidence, considering time and sex as covariates.

**Results:**

In 2023, Argentina exhibited an age‐standardized incidence of 25.3 new dementia cases per 100,000 inhabitants (19.1 for men and 31.1 for women). (Table 1, Figure 1) A significant increase in incidence (p = 0.029) was observed over the 10 years (yearly effect: beta = 0.14, 95% CI [0.02, 0.26]) (Figure 2), with no significant sex‐specific impact on incidence evolution (p = 0.8). Early‐onset dementia reported an incidence of 5 per 100,000, contrasting with late‐onset dementia at 26.1. Late‐onset dementia displayed significant growth compared to early‐onset cases (p = 0.07).

**Conclusion:**

The increasing dementia incidence in Argentina emphasizes the urgent need for targeted interventions. Although there is no differential growth in incidence between men and women, women consistently exhibit a higher occurrence. Early‐onset dementia, traditionally associated with genetic predisposition, has remained stable. In contrast, late‐onset dementia has surged recently, likely due to the aging of the population and the rise in modifiable risk factors reported by other regional studies. These findings serve as a call to action, emphasizing significant regional prevention potential.